# The landscape of biased agonism in aryl hydrocarbon receptor signaling: current clues and future directions

**DOI:** 10.3389/fphar.2026.1823615

**Published:** 2026-05-14

**Authors:** Zdeněk Dvořák, Jakub Přikryl

**Affiliations:** Department of Cell Biology and Genetics, Faculty of Science, Palacký University, Olomouc, Czechia

**Keywords:** AhR receptor, biased agonism, cellular signaling, functional selectivity, non-canonical signaling pathway

## Abstract

Our understanding of the aryl hydrocarbon receptor (AhR) signaling pathway has significantly advanced since its initial discovery, revealing roles in both toxicology and normal physiological processes like immunity and organ development. However, key gaps remain concerning the precise mechanisms governing ligand-dependent canonical and non-canonical signaling. From a pharmacological standpoint, this involves the concept of functional selectivity, also known as biased agonism, where different ligands can selectively activate distinct downstream pathways, leading to different biological or toxicological outcomes. This paper reviews the current understanding of the formation, composition, and function of both cytosolic and nuclear AhR multiprotein complexes. We analyze the early and late cellular events that follow AhR ligand binding and explore new approaches aimed at the selective targeting of the AhR canonical and non-canonical signaling pathways for potential therapeutic benefit.

## Introduction

The aryl hydrocarbon receptor (AhR) operates through two primary modes of action: genomic and non-genomic signaling (Sondermann et al., 2023; Coumoul et al., 2026). The latter comprises mechanisms such as E3 ubiquitin ligase activity (Ohtake et al., 2007) or the indirect activation of the EGF receptor via c-Src kinase (Vogeley et al., 2022). Within its genomic signaling framework, AhR regulates the expression of gene expression through both canonical, and non-canonical pathways (Coumoul et al., 2026). These two pathways differ in several key aspects. Regarding target genes, AhR-regulated genes are referred to as canonical (e.g., *CYP1A1*) or non-canonical (e.g., *PAI-1*). In terms of DNA binding, AhR interacts with canonical (xenobiotic-responsive elements; XREs) or non-canonical (non-XREs) gene promoters. Furthermore, nuclear AhR forms transcriptionally active heterodimers with either canonical (AhR nuclear translocator; ARNT) or non-canonical (e.g., Krüppel-like factor 6; KLF6) protein partners.

Thus, canonical vs*.* non-canonical AhR signaling is well-defined at the level of nuclear protein partners, DNA promoters, and target genes. However, the upstream events in AhR cellular signaling that distinguish between these pathways remain unknown. A pivotal question remains: what is the initial driving factor governing the selection between canonical and non-canonical AhR signaling? Deciphering the mechanisms underlying AhR functional selectivity might open new avenues for the selective targeting of these pathways.

## Assembly and structure of the AHR protein cytosolic complex

The conventional description of AhR cellular signaling is based on the primary assumption that these are two distinct cellular states: the absence or the presence of a ligand. Accordingly, the first step in AhR signaling is commonly described as follows: “*In its resting state, the unliganded AhR resides in the cytoplasm in a complex with chaperone proteins, such as hsp90, XAP2, p23, and others. The role of these protein partners are: (i) to mask nuclear localization signal (NLS) sequence at the N-terminus; (ii) to maintain AhR in a conformation conductive to binding; (iii) to protect AhR from proteasomal degradation.*”

Knowledge regarding the events preceding the formation of the cytosolic AhR protein complex - and the involvement of putative auxiliary proteins specific for the assembly of the AhR complex in canonical and non-canonical pathways - remains limited. However, roles for common players such as hsp70 and Hop (STIP1), in the process of loading AhR onto hsp90 have been proposed (Wen et al., 2023).

The composition of the cytosolic AhR multiprotein complex (*AhRCyt*) has been studied for over 3 decades. *Heat shock protein 90 kDa (hsp90)*: Initially, Perdew demonstrated that the *AhRCyt* (in Hepa1c1c7 cells) has a tetrameric structure, and that the AhR protein partners are approximately 85 kDa in size (Perdew, 1992). In follow-up studies, Perdew identified hsp90 as a component of *AhRCyt*. The hsp90/AhR stoichiometry was determined to be 2.4 and 1.7, depending on the technique used. The presence of an unknown 43 kDa protein in *AhRCyt* was also reported (later identified as XAP2). Interestingly, specific antibodies targeted against mouse hsp84 and hsp86 detected both isoforms (corresponding to human hsp90α and hsp90β) (Chen and Perdew, 1994). Ogiso *et al* studied the phosphorylation of hamster hsp90 in complex with mAhR, reporting the presence of both hsp90α and hsp90β isoforms in *AhRCyt* (Ogiso et al., 2004). Different hsp90 isoforms were also identified in a complex of yeast hsp90 with hAhR (Cox and Miller, 2003). *HBV X-Associated Protein 2 (XAP-2)*: The interaction of XAP-2 with mAhR and hsp90 was identified *via* yeast two-hybrid assays and co-immunoprecipitation in reticulocyte lysates (Carver and Bradfield, 1997) and mouse hepatoma cells (Ma and Whitlock, 1997). These interactions were further characterized by Perdew (Meyer and Perdew, 1999). Interestingly, in monkey COS-1 cells co-transfected with AhR and XAP-2, 33% less XAP-2 co-immunoprecipitated with hAhR than with mAhR, while the amount of hsp90 remained similar between the two receptors (Ramadoss et al., 2004). Residue Tyr408 of AhR was demonstrated to be essential for the interaction with XAP-2. Furthermore, XAP-2 is capable of forming multimeric complexes independently of hsp90 or AhR, though it is not yet clear if XAP-2 exists within the AhR complex in more than one copy (Hollingshead et al., 2004).

Recent advances in cryo-electron microscopy (cryo-EM) have opened new avenues for studying the *AhRCyt*. Gruszczyk *et al* described the cryo-EM structure of a liganded quinary *AhRCyt*, having a composition: hAhR (271–427)-hsp90β-hsp90β-XAP2-indirubin (pdb: 7ZUB). They performed co-expression of recombinant proteins in insect Sf9 cells. They also reported several hsp90-XAP2-AhR-p23 complexes that varied in the number and location of the p23 protein (Gruszczyk et al., 2022). The cryo-EM structural data were largely reproduced by Wen *et al* in a study of unliganded mAhR, which identified two types of *AhRCyt*: a quinary complex mAhR (268–407)-hsp90β-hsp90β-p23-p23 and a senary complex mAhR (269–278)-hsp90β-hsp90β-p23-p23-XAP2 (Wen et al., 2023). Notably, further structural studies using full-length endogenous hAhR and its partner proteins are still required.

A lingering question is whether a single endogenous *AhRCyt* exists or if a variety of complexes are formed? Could distinct complexes - differing in the presence, spatial arrangement, or stoichiometry of partner proteins (including their isoforms) – determine the AhR preference for canonical vs*.* non-canonical signaling? Narita *et al* reported different liganded *AhRCyt* compositions, depending on the ligand type in mice brains and human HeLa cells. The toxic xenobiotic ligand 3-methylcholanthrene (3 MC) bound to *AhRCyt* containing hsp90, XAP-2 and p23, whereas the non-toxic microbial-derived ligand 1,4-dihydroxy-2-naphthoic acid (DHNA) bound to a complex *AhRCyt* containing hsp90 and XAP-2, but lacking p23 (Narita et al., 2024). Furthermore, the 3 MC-AhR-hsp90-XAP2-p23 complex translocated to the nucleus rapidly (<30 min) and strongly induced the xenobiotic-related gene *CYP1A1*, while the DHNA-AhR-hsp90-XAP2 complex translocated more slowly (>60 min) and induced *CYP1A1* only weakly. These data suggest biased agonism, where two different ligands result in qualitatively different *AhRCyt* with quantitatively different functions. However, it remains to be determined whether different ligands select for pre-existing distinct *AhRCyt* or if the ligands themselves transform an universal *AhRCyt* into different forms (*vide infra*).

## Binding of ligands to AHR

**FIGURE 1:**
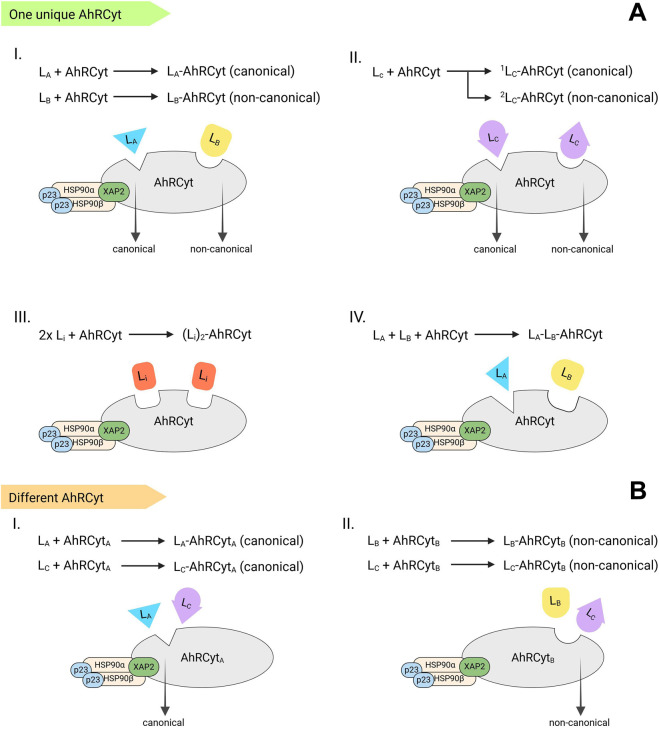
Scenarios of ligand-receptor interactions **(A)** Single *AhRCyt* form (I.) Ligands *L*_*A*_ and *L*_*B*_ trigger canonical (*L*_*A*_-*AhRCyt*) and non-canonical (*L*_*B*_-*AhRCyt*) signaling, respectively, by adopting distinct binding modes within *AhRCyt* (II.) The ligand *L*_*C*_ interacts with *AhRCyt* through two different binding modes, enabling the receptor to activate either canonical (^*1*^ *L*_*C*_ *-AhRCyt*) or non-canonical (^*2*^ *L*_*C*_ *-AhRCyt*) pathways (III.) Concomitant binding of two identical small molecules to the PAS-A domain, exhibiting a 2:1 (*L*_*i*_)_*2*_-*AhRCyt* stoichiometry (IV.) Concomitant binding of two different small molecules to the PAS-A domain, exhibiting 2:1 *L*_*A*_-*L*_*B*_-*AhRCyt* stoichiometry **(B)** Multiple *AhRCyt* variants: Ligands *L*_*A*_ and *L*_*B*_ bind to specific receptor complexes *AhRCyt*_*A*_ and *AhRCyt*_*B*_, respectively, triggering canonical or non-canonical signaling via the resulting ligand-receptor complexes *L*_*A*_-*AhRCyt*_*A*_ and *L*_*B*_-*AhRCyt*_*B*_. Ligand *L*_*C*_ mediates either pathway by interacting with different *AhRCyt* variants to form *L*_*C*_-*AhRCyt*_*A*_ and *L*_*C*_-*AhRCyt*_*B*_.

The next step is defined as follows: “*The binding of a ligand induces a conformational change of the AhR, which results in the unmasking of the NLS.*” This suggests that the early biochemical event (transduction), occurring upon ligand binding (recognition) is a conformational change of the AhR protein or the entire *AhRCyt*. Several scenarios of ligand-receptor interactions might be conceived (Figure 1):

- Assuming the existence of a single *AhRCyt* form: In this scenario, the chemical structure of a ligand (*L*) solely determines its unique interaction with amino acid residues in the AhR PAS-B ligand binding domain (recognition) (Giani Tagliabue et al., 2019), leading to a ligand-specific conformational change of *AhRCyt.* Consequently, this change is transduced into qualitative (functional selectivity, biased agonism) and quantitative (intrinsic activity, agonism-antagonism) receptor responses. This implies that ligands *L*_*A*_ and *L*_*B*_ trigger canonical (*L*_*A*_-*AhRCyt*) and non-canonical (*L*_*B*_-*AhRCyt*) signaling, respectively, through distinct binding modes within *AhRCyt.* Accordingly, it is conceivable that a single ligand *L*_*C*_ might interact with *AhRCyt* in two different modes (^*1*^ *L*_*C*_ *-AhRCyt/* ^*2*^ *L*_*C*_ *-AhRCyt*), enabling the AhR to activate canonical and/or non-canonical pathways. Additional complexity arises from the potential concomitant binding of two small molecules in AhR PAS-A. Hubbard *et al* reported that the human AhR, acting as a host indole receptor, may exhibit a unique (*L*_*i*_)_*2*_-*AhRCyt* stoichiometry (Hubbard et al., 2015). Similarly, Delfosse *et al* established the concept of a “supramolecular ligand” for the AhR-sister pregnane X receptor (PXR), demonstrating that the ligand *L*_*A*_ and *L*_*B*_ - both exhibiting low efficacy individually - cooperatively bind to PXR, to induce synergistic activation (Delfosse et al., 2015).
- Assuming the existence of multiple *AhRCyt* isoforms: In this case, both the ligand (*L*) and the specific receptor form (*AhRCyt*) determine the formation of unique ligand-receptor *L-AhRCyt* complexes. Beyond the single-form *L-AhRCyt* interactions mentioned above, qualitatively different *L-AhRCyt* interactions may occur if multiple variants of *AhRCyt* are available. It is plausible that the affinity-driven preferential binding of ligands (*L*) to the specific forms of *AhRCyt* takes place. This implies that ligands *L*_*A*_ and *L*_*B*_ bind to receptors *AhRCyt*_*A*_ and *AhRCyt*_*B*_, respectively, thereby triggering canonical and non-canonical signaling *via* ligand-receptor complexes *L*_*A*_-*AhRCyt*_*A*_ and *L*_*B*_-*AhRCyt*_*B*_ *.* Furthermore, one ligand *L*_*C*_ could mediate both signaling types by forming distinct complexes *L*_*C*_-*AhRCyt*_*A*_ and *L*_*C*_-*AhRCyt*_*B*_ *.*

A growing body of evidence shows that different ligands bind the AhR PAS-B domain by interacting with distinct amino acids residues. Giani-Tagliabue *et al* predicted and experimentally validated specific residues within the mAhR binding cavity that play a critical role in the binding of three distinct chemical groups (Giani Tagliabue et al., 2019). Group I ligands, including 2,3,7,8-tetrachlorodibenzo-*p-*dioxin (TCDD), 2,3,7,8-tetrachlorodibenzofuran, and benzo [*a*]pyrene (BaP), bind at the bottom of the cavity (Bβ, Cα, Dα region) and are stabilized by hydrophobic interactions. Group II ligands, such as 3MC, PCB126, and dibenz [*a*,*h*]anthracene, bind nearer to the Fα-Met334/Gβ-Met342 site and also have hydrophobic interactions. Group III ligands (e.g., indirubin, leflunomide, β-naphtoflavone BNF, 6-Formylindolo [3,2-*b*]carbazole FICZ), occupy a similar position to Group II (Fα-Cys327,Ser330/Gβ-Met342), but establish hydrogen-bonds and polar interactions with Gln377 and Ser359 (Giani Tagliabue et al., 2019).

The first structure of a ligand-bound *AhRCyt* was resolved by Gruszczyk *et al* using cryo-EM analysis of a complex hAhR (271–427)-hsp90β-hsp90β-XAP2-indirubin (pdb: 7ZUB) (Gruszczyk et al., 2022). They identified residues essential for the interacting with the indole rings I (Phe295, Tyr322, Ile325, Cys333, His337, Ile349, Phe351) and II (Ser365 a Q383 + His291, Pro297, Gly321, Phe324, Phe351, Leu353, Ser365, Val381, Gln383) of the indirubin molecule. In a follow-up study, the cryo-EM structure of the complex bound to BaP was solved (pdb: 8QMO) (Kwong et al., 2024). Superimposition of the BaP- and indirubin-bound AhR-Hsp90-XAP2 complexes revealed largely overlapping binding sites; however, indirubin formed two additional electrostatic interactions with Ser365 and Gln383. These stable hydrogen bonds likely explain the two-orders-of-magnitude higher affinity of indirubin compared to BaP.

Diao *et al* bacterially co-expressed porcine pAhR (26–414) and hARNT (85–465), obtaining crystals with 21-mer double-stranded DNA and six different AhR ligands (pdb codes: tapinarof-8XS6, FICZ-8XS7, BaP-8XS8, BNF-8XS9, indigo-8XSA, indirubin-8XSB) (Diao et al., 2025). Cell-based *in vitro* approaches and X-ray analysis identified twenty-one pAhR residues crucial for ligand interaction. Eight residues (His289, Phe293, Gly319, Cys331, Phe349, Leu351, Ser363, Gln381) define the general outline of the binding pocket, while others provide a unique interaction network for specific ligands. For instance, Tyr334 and Ser344 interact exclusively with FICZ, whereas Tyr320 and His335 interact only with indirubin. Pro295 is specific to tapinarof and BNF, while Ile347 is essential for all studied ligands except BaP (Diao et al., 2025).

## AHR nuclear translocation

“*Upon binding a ligand, the L-AhRCyt translocates to the cell nucleus*.” The nuclear localization signal (NLS) is bipartite located within the *N*-terminus (_13_RKRRK_17_) and bHLH domain (_37_KR-R_40_) of the AhR (Ikuta et al., 1998). Mutation studies in MCF-7 cells revealed that the first sequence _13_RKRRK_17_ is essential for ligand-inducible nuclear translocation, whereas the second sequence _37_KR-R_40_ plays a supportive role in modulating translocation speed (Haidar et al., 2023). In this study, the authors also demonstrated the effects of specific inhibitors of importins alpha and beta1, concluding that ligand-induced and basal nuclear entry rely on the same mechanism but are uniquely controlled by the two NLS components. They further speculated that difference in the speed and intensity of translocation depend on the degree of NLS masking by hsp90. This is consistent with previous observations that hsp90 interacts with the bHLH region of unliganded *AhRCyt*, and that subsequent ligand binding induces a conformational change that unmasks NLS (Ikuta et al., 1998; Kudo et al., 2018).

However, regardless of the presence or absence of a ligand, AhR is distributed between the cytosol and the nucleus; thus, it is not exclusively localized in either compartment. A solid body of evidence from the Perdew lab attests to the role of XAP-2 in AhR sub-cellular localization and function. While transiently expressed mAhR typically localizes to the nucleus, co-expression with XAP-2 restored its cytoplasmic localization–an effect that was reversed by TCDD. The portion of endogenous mAhR associated with XAP2 correlated with its distribution between the cytoplasm and nucleus. Furthermore, the study suggested that ligand binding initiates nuclear translocation prior to complex dissociation. Although the existence of two structurally and potentially functionally distinct forms of mAhR has been demonstrated (Petrulis et al., 2000), their association with canonical or non-canonical signaling remains unestablished. Notably, the inhibition of ligand-independent shuttling by XAP-2 was not caused by physical tethering or NLS blockage. Instead, XAP-2 hindered the binding of importin beta to the mAhR complex, suggesting that XAP2 alters the conformation of the bipartite NLS. XAP-2 also repressed the transactivation potential of mAhR, likely by stabilizing the receptor complex or blocking its nucleocytoplasmic shuttling (Petrulis et al., 2003). In contrast, results for hAhR suggest it differs biochemically from mAhR. Transiently expressed hAhR localizes predominantly in the cytoplasm; its localization was unaffected by XAP-2 co-expression and was not blocked by leptomycin B. While co-expression of mAhR with XAP2-NLS prompted cytoplasmic localization, hAhR remained partially nuclear, suggesting that XAP-2 stays bound to hAhR during shuttling (Ramadoss et al., 2004). Finally, two protein kinase C sites (Ser12, Ser36) were identified upstream from each NLS, where phosphorylation status governs subcellular localization. This supports a proposed two-step mechanism for ligand-dependent nuclear translocation of hAhR (Ikuta et al., 2004).

## Assembly and structure of the AHR protein nuclear complex

It is recognized that “*upon reaching the cell nucleus, AhR partner proteins dissociate from L-AhRCyt, the nuclear complex L-AhRNuc is formed, and it, in turn, it binds to the promoters of AhR-target genes*”.

**FIGURE 2:**
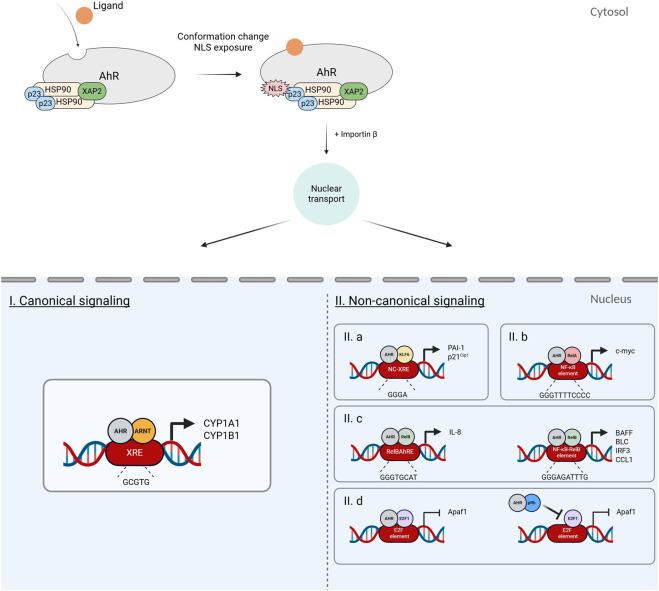
Canonical *vs*. non-canonical AhR signaling. Differential recruitment of nuclear protein partners and binding to distinct gene regulatory elements.

In the canonical AhR signaling pathway, *AhRNuc* exists as an AhR-ARNT heterodimer (Figure 2). The structures of crystallized AhR-ARNT complexes have been elucidated by several research groups: (i) mAhR-PAS-A (110–267) + mARNT (pdb: 4M4X) (Wu et al., 2013); (ii) mAhR (PAS-A, bHLH) + hARNT + DNA (XRE) (pdb: 5V0L) (Seok et al., 2017); (iii) hAhR (23–273) + mARNT + DNA (XRE) (pdb: 5NJ8) (Schulte et al., 2017); (iv) pAhR (26–414) + hARNT + DNA (XRE) + L (six different ligands) (Diao et al., 2025). The latter study explains the dynamic pAhR conformation shifts during the transition between unliganded *AhRCyt*, liganded *L-AhRCyt*, and liganded *L-AhRNuc*. They show that indirubin binding induces the outward movement of Val348 and Phe349, which facilitates the formation of a hydrogen bond between Val348 and Arg396, and a salt bridge between Arg396 and Asp327 (Diao et al., 2025). Hence, it is assumed that different ligands (*L)* interact with different sets of amino acids residues in the AhR PAS-B domain, putatively engaging different *L-AhRCyt* conformations. Thereby, distinct *L-AhRCyt* are formed and eventually transformed into different *L-AhRNuc* AhR-ARNT complexes. Ultimately, *L-AhRNuc* binds consensus XRE elements in the promoters of AhR-target genes. It may be speculated that different *L-AhRNuc* complexes bind preferentially to XREs in different gene promoters. Based on this mechanism, functional selectivity might be mapped onto the axis involving four structural and functional states of AhR: *AhRCyt* → *L-AhRCyt* → *L-AhRNuc* → *L-AhRNuc-XRE*. Diao *et al* also showed that the spatial location of the ARNT PAS-B domain does not directly conflict with hsp90 or XAP-2, suggesting the existence of a transitional state (*L-AhRCyt* → *L-AhRNuc*) where ARNT can directly engage with AhR while it is still bound to hsp90 and XAP-2. Binding of ARNT may ultimately displace XAP-2 from *L-AhRNuc* (Diao et al., 2025). Of note, human ARNT exist in two isoforms, ARNT1 and ARNT2. All abovementioned studies refer to ARNT1. Since the expression of ARNT2 has been reported only in the brain and skeletal muscle (Barrow et al., 2002; Imran et al., 2015), ARNT1 is considered the primary player in the assembly of *L-AhRNuc* in most tissues.

Non-canonical AhR signaling is defined by the formation of an *L-AhRNuc* involving proteins other than ARNT, and the binding of *L-AhRNuc* to non-consensus NC-XREs in non-canonical genes (Figure 2). Examples of non-canonical genes include those encoding plasminogen activator inhibitor-1 (PAI-1) (Huang and Elferink, 2012), apoptotic protease activating factor-1 (Apaf1) (Marlowe et al., 2008), interleukin 8 (IL-8) (Vogel et al., 2007a), CC-motif Chemokine Ligand-1 (CCL1) (Vogel et al., 2007b), and p21^Cip1^ genes (Jackson et al., 2014). Several non-canonical partners in *L-AhRNuc* have been identified, including KLF6 (Jackson et al., 2014; Wilson et al., 2013), retinoblastoma protein pRb (Ge and Elferink, 1998; Puga et al., 2000), E2F1 (Marlowe et al., 2008), RelA (Tian et al., 1999; Kim et al., 2000; Chen et al., 2012), and RelB (Vogel et al., 2007a; Vogel et al., 2007b). Interactions of AhR with other proteins, such as signal transducer and activator of transcription 1 (STAT1) (Kimura et al., 2009; Kimura et al., 2008), Src kinase (Xie et al., 2012), and estrogen receptor alpha (ERα) (Klinge et al., 2000), have been reported, but are not classically categorized as non-canonical signaling (*cf.* introduction). Nevertheless, it remains unclear which factor dictates the selection of partner protein engaged in *L-AhRNuc*, thereby determining AhR functional selectivity. Studies on non-canonical partners of liganded nuclear AhR have been largely restricted to using TCDD as the AhR ligand (Ovrevik et al., 2014). Hence, one ligand (TCDD; *L*_*C*_) is capable of triggering both canonical and non-canonical signaling by recruiting different partner proteins to *L*_*C*_ *-AhRNuc*. If only one *AhRCyt* form exists, then TCDD (*L*_*C*_) must interact with different residues in AhR PAS-B, resulting in the formation of two distinct complexes, ^*1*^ *L*_*C*_ *-AhRCyt* and ^*2*^ *L*_*C*_ *-AhRCyt* (Figure 1A). Alternatively, if multiple *AhRCyt* forms exist, the binding of TCDD (L_C_) to *AhRCyt*_*A*_ or *AhRCyt*_*B*_ leads to the formation of complexes *L*_*C*_ *-AhRCyt*_*A*_ and *L*_*C*_ *-AhRCyt*_*B*_ (Figure 1B). These different *L-AhRCyt* (^*1*^ *L*_*C*_ *-AhRCyt/* ^*2*^ *L*_*C*_ *-AhRCyt* or *L*_*C*_ *-AhRCyt*_*A*_/*L*_*C*_ *-AhRCyt*_*B*_) forms are then transformed into specific *L*_*C*_ *-AhRNuc* complexes, conferring canonical or non-canonical signaling.

## Biased signaling in targeting the AHR

The concept of functional selectivity or biased agonism has become well-established in receptor pharmacology, particularly with G protein-coupled receptors (GPCRs), and is now a critical area of study for the AhR. Biased signaling refers to the ability of a specific ligand to selectively activate certain downstream signaling pathways over others when compared to a different ligand. The diverse physiological outcomes and toxicological effects of the AhR are highly dependent on the specific ligand that stimulates it. The molecular mechanisms underlying this phenomenon include.

### Differential conformational changes

Different ligands bind within the AhR ligand-binding domain using unique sets of amino acid residues. This induces distinct conformational changes in the AhR protein or the entire cytosolic AhR complex. Recently reported cryo-EM structures of liganded mouse (Wen et al., 2023) and human (Gruszczyk et al., 2022; Kwong et al., 2024) AhR cytosolic complexes, along with X-ray crystal structures of porcine AhR-ARNT nuclear complexes (Diao et al., 2025), have significantly advanced our understanding of how different ligands engage specific amino acids within the AhR protein to yield distinct molecular structures. However, further studies on the interactions between small-molecule ligands and the AhR protein are warranted, as existing data present several limitations: (i) Interspecies differences: Significant variations in affinity and activity have been reported for several AhR ligands, such as indoles (Hubbard et al., 2015) and polyaromatic hydrocarbons (Vondracek et al., 2017); therefore, a focus on the human AhR is essential. (ii) Construct length: Data from truncated AhR may not fully reflect the behavior of the full-length protein. Attempts to use longer AhR constructs are needed to address this. (iii) Limited scope: Only a few complexes and ligands have been studied to date. A robust and high-throughput experimental approach would be highly beneficial.

### Distinct complex formation

Different ligands can lead to the formation of qualitatively and quantitatively distinct ligand-AhR complexes, which hold different functions. For example, the toxic ligand 3 MC forms a complex containing the p23 protein that rapidly translocates to the nucleus and strongly induces the canonical gene *CYP1A1*, whereas the non-toxic ligand DHNA forms a complex without p23 that translocates slower and induces the same gene only weakly. The composition of cytosolic and nuclear AhR multiprotein complexes has traditionally been studied using classical molecular biology and biochemistry. Identification typically relied on immunochemiluminiscence following SDS-PAGE, methods often limited by their sensitivity and robustness. In contrast, modern shotgun proteomics (LC-MS/MS) offers a powerful platform for resolving the structures of *AhRCyt*, *L-AhRCyt, AhRNuc*, and *L-AhRNuc* complexes, enabling the identifications of ligand and protein patterns that define canonical and non-canonical pathways.

### Selective partner recruitment

The specific complex formed in the cytoplasm is eventually transformed into different nuclear complexes that recruit different nuclear protein partners. While the canonical pathway involves dimerization with ARNT, non-canonical pathways involve partners such as KLF6, pRb, E2F1, RelA, and RelB. The specific partner recruited dictates the functional outcome and whether the complex binds to canonical XREs or non-canonical NC-XREs promoters. Modern technologies such as Chromatin Immunoprecipitation Sequencing ChIP-Seq, are powerful methods for mapping genome-wide protein-DNA binding sites (Shahriar et al., 2026). Furthermore, techniques like RNA-seq, DNA microarrays, and high-throughput qPCR allow for robust profiling of AhR-regulated gene expression. In combination with bioinformatic tools, these expression patterns can be correlated with AhR agonist structures, the composition of cytosolic and nuclear AhR multiprotein complexes, and AhR binding at DNA promoters. Collectively, these data map the landscape canonical and non-canonical AhR signaling.

Understanding AhR biased signaling has significant implications for drug development. The goal is to identify and develop selective AhR modulators that induce beneficial outcomes (e.g., immunomodulation, anti-cancer effects) without causing the major toxic outcomes associated with global activators like TCDD. Novel therapeutic strategies targeting the AhR leverage recent structural breakthroughs, including cryo-electron microscopy and X-ray crystallography data, to: (i) Develop ligands that selectively activate specific pathways. (ii) Target the AhR’s function in immune responses and cancer therapy. (iii) Utilize small molecules or peptidomimetics to disrupt specific protein-protein or protein-DNA interactions, potentially inhibiting only the canonical or non-canonical pathways.
